# Editorial: Molecular neurobiology of chronic pain: pharmacological and non-pharmacological approaches in animal models

**DOI:** 10.3389/fnmol.2024.1487141

**Published:** 2024-09-16

**Authors:** Mario A. Acuña, Kristina Valentinova, Pascal Darbon

**Affiliations:** ^1^Department of Physiology, University of Bern, Bern, Switzerland; ^2^Department of Neuroscience, Physiology and Pharmacology, University College London, London, United Kingdom; ^3^Equipe Contrôle Peptidergique des Émotions Institut des Neurosciences Cellulaires et Intégratives, Strasbourg, France

**Keywords:** gene therapy, chronic pain, non-pharmacological treatment, migraine, natural compounds

Chronic pain remains a considerable global health challenge, often resistant to conventional treatments. This Research Topic in Frontiers in Molecular Neuroscience showcases cutting-edge studies exploring innovative approaches to chronic pain management in rodent models. From pioneering gene therapy techniques to treat osteoarthritis to detailed analyses of molecular pathways in migraine, investigations into natural compounds for neuropathic pain, and a comprehensive review of migraine pathophysiology, these studies represent the forefront of pain research. Together, they offer new insights into the mechanisms underlying chronic pain conditions and potential strategies for more effective management, particularly in the realm of migraine and neuropathic pain, and osteoarthritis.

The work of Zhuang et al. and Kandel et al. demonstrates the potential of gene therapy in addressing chronic pain. Their research on carbonic anhydrase-8 (CA8) gene therapy using replication-defective herpes simplex virus (rdHSV) vectors offers a promising non-opioid approach to pain management. Zhuang et al. demonstrated that a novel rdHSV-CA8 effectively treats chronic osteoarthritis knee pain induced by monoiodoacetate (MIA). Their findings show that intra-articular knee joint administration of rdHSV-vHCA8 produces significant analgesia in this model. Importantly, they identified that this analgesic effect is mediated through the activation of Kv7 voltage-gated potassium channels, providing a mechanistic understanding of the treatment's efficacy. Complementing these findings, Kandel et al. further elucidated the cellular mechanisms underlying the analgesic effects of rdHSV-CA8 gene therapy. Through whole-cell patch-clamp recordings, they showed that vHCA8 transduction in small dorsal root ganglion (DRG) neurons causes prolongation of their afterhyperpolarization (AHP), a key regulator of neuronal excitability. This effect was completely reversed by the specific Kv7 channel inhibitor XE-991, confirming the role of Kv7 channels in mediating the analgesic effect. Together, these studies provide evidence for the efficacy of CA8 gene therapy in reducing neuronal excitability and producing analgesia through a Kv7 channel-dependent mechanism. This non-opioid approach offers an alternative for chronic pain management, potentially avoiding the pitfalls associated with traditional opioid-based treatments.

Another contribution to this Topic comes from Zhang Z.-L. et al., where they investigated the potential of Tetrandrine (TET) in alleviating oxaliplatin-induced neuropathic pain, addressing a critical issue in cancer treatment: chemotherapy-induced peripheral neuropathy. The study demonstrated that TET, a bisbenzylisoquinoline alkaloid extracted from Stephania tetrandra S. Moore, effectively reduces mechanical allodynia in a rat model of oxaliplatin-induced neuropathic pain. The researchers found that TET administration significantly reduced mechanical allodynia in oxaliplatin-treated rats. They identified several inflammation-related genes (IRGs) that were modulated by TET, including Arg2, Cxcl12, H2-Q6, Kdr, and Nfkbia. These findings suggest that TET's analgesic effects are mediated through the modulation of inflammatory pathways. The study's exploration of immune infiltration in neuropathic pain provides new insights, suggesting a link between Tetrandrine's effects and immune response modulation. The authors observed that oxaliplatin treatment increased follicular CD4 T cell infiltration, which was subsequently reduced by TET administration. Finally, the authors' use of molecular docking studies to predict the binding of Tetrandrine. The docking studies revealed strong binding affinities between TET and proteins encoded by Arg2, Cxcl12, Kdr, and Nfkbia, further supporting the proposed mechanism of action. This comprehensive study not only sheds light on the potential of Tetrandrine as a novel treatment for chemotherapy-induced neuropathic pain but also provides a multi-faceted approach to understanding its mechanism of action, from gene modulation to immune response, paving the way for more targeted and effective therapies in the future.

The study by Zhang X. et al. provides insights into the role of SIRT1 in chronic migraine, a regulator of mitochondria dynamics-related proteins, highlighting the importance of understanding underlying molecular mechanisms in developing targeted therapies. Their work suggests a role of SIRT1 in generating reactive oxygen species (ROS) and NMDAR2B phosphorylation opening new avenues for exploring non-pharmacological interventions in migraine treatment. The study's exploration of the SIRT1-ROS-NMDAR2B pathway provides a comprehensive view of the molecular mechanisms involved in chronic migraine. By employing a combination of pharmacological interventions (SIRT1 activator SRT1720, SIRT1 inhibitor EX527, and ROS scavenger tempol), the authors demonstrated the regulatory role of SIRT1 in ROS production and its downstream effects on NMDAR2B phosphorylation and central sensitization. This mechanistic insight advances our understanding of chronic migraine pathophysiology and identifies potential targets for therapeutic intervention.

Finally, Frimpong-Manson et al. offer a comprehensive review of migraine pathophysiology, bridging the gap between preclinical research and clinical applications. Their analysis of various animal models and potential therapeutic targets, including the endocannabinoid system and TRP channels, provides direction for future research in non-pharmacological pain management strategies. A key aspect of the Frimpong-Manson et al. review is its analysis of animal models used in migraine research. The authors evaluate various preclinical models, including those based on cortical spreading depression, nitroglycerin-induced hyperalgesia, and inflammatory mediator application. Their discussion of the strengths and limitations of each model offers guidance for researchers in selecting appropriate experimental paradigms for their specific research questions. Additionally, by contextualizing animal study results within the framework of human migraine presentations, the authors provide a perspective on the translational potential of various research findings. This approach is crucial for guiding the development of more effective and targeted migraine therapies.

In conclusion, this Research Topic highlights the ongoing advancements in pain research. By exploring non-pharmacological interventions, particularly gene therapy, and providing the basis for potential pharmacological treatment it uncovers new possibilities for improving the lives of those suffering from chronic pain. The work presented here advances our scientific understanding and offers potential for more effective, safer pain management strategies in the future.

